# Indication, technical considerations, and outcome of remote central cannulation for repeat extracorporeal membrane oxygenation in congenital diaphragmatic hernia: a case report

**DOI:** 10.1051/ject/2025019

**Published:** 2025-09-15

**Authors:** Enrico Danzer, Sabrina J. Flohr, Holly L. Hedrick, Geoffrey L. Bird, Jonathan M. Chen, Natalie E. Rintoul

**Affiliations:** 1 The Richard Wood Jr. Center for Fetal Diagnosis and Treatment, The Children’s Hospital of Philadelphia, 5th Floor Wood Center, The Children’s Hospital of Philadelphia 3615 Civic Center Blvd Philadelphia PA 19104-4318 USA; 2 Division of Critical Care, Department of Anesthesiology and Critical Care Medicine, Cardiac Center at the Children’s Hospital of Philadelphia, Division of Cardiology, Department of Pediatrics, Cardiac Center at the Children’s Hospital of Philadelphia 3615 Civic Center Blvd Philadelphia PA 19104-4318 USA; 3 Division of Cardiothoracic Surgery, Cardiac Center at the Children’s Hospital of Philadelphia 3615 Civic Center Blvd Philadelphia PA 19104-4318 USA

**Keywords:** Congenital diaphragmatic hernia, Extracorporeal membrane oxygenation (ECMO), Central cannulation, Multicourse ECMO, Pulmonary hypertension

## Abstract

Repeat extracorporeal membrane oxygenation (ECMO) is rare in children with congenital diaphragmatic hernia (CDH). Improving our understanding of the potential survival benefits, complications, and surgical challenges associated with this procedure is essential for enhancing decision-making regarding multicourse ECMO in CDH. We report the case of a now 3-year-old girl who required cannulation through median sternotomy 5 months after her initial neonatal ECMO treatment via cervical venoarterial cannulation. This second run of ECMO was performed due to an acute exacerbation of pulmonary hypertension caused by urosepsis. This case illustrates that repeat ECMO should be considered for selected CDH patients when a reversible cause for clinical deterioration is identified. We also emphasize the importance of interdisciplinary decision-making, considering alternative cannulation methods, and providing appropriate family counseling. It is crucial to balance the potential survival benefits of repeat ECMO against the increased risks of morbidity.

## Introduction

Neonates with congenital diaphragmatic hernia (CDH) complicated by severe pulmonary hypoplasia, pulmonary hypertension, and cardiac dysfunction may require extracorporeal membrane oxygenation (ECMO) to support them through the initial cardiorespiratory transition and to provide an opportunity for surgical repair [1, 2]. While approximately one-third of newborns with CDH receive ECMO as a life-sustaining intervention in the neonatal period, the utilization of multicourse ECMO in this population is rare [3]. Therefore, data on recannulation needs and outcomes are scarce, limiting our understanding of the effects of multicourse ECMO on neonates with CDH.

Herein, we report a case of a now 3-year-old female who required repeat cannulation via a median sternotomy 5 months after the initial neonatal ECMO cervical venoarterial run due to an acute exacerbation of pulmonary hypertension secondary to urosepsis. This case demonstrates that repeat ECMO represents a potential management strategy for selected CDH patients in which a reversible cause for clinical deterioration can be identified. We also highlight the interdisciplinary decision-making process, the importance of considering alternative cannulation approaches, and the need for appropriate family counseling, as the potential survival benefits of repeat ECMO must be balanced against the increased risks of morbidities.

## Case report

This case involves a now 3-year-old African American female who was diagnosed with a left-sided congenital diaphragmatic hernia (CDH) at 21 weeks and 2 days of gestation. At that time, her observed-to-expected lung head ratio (O/E LHR) was 25.1% (using the trace method), and her observed-to-expected total lung volume (O/E TLV) was 25.4% (using Meyer’s method [4]. Additionally, there was evidence of an intrathoracic liver position. The low O/E LHR and O/E TLV, combined with the intrathoracic position of the liver, were indicative of severe pulmonary hypoplasia, known to be associated with significant neonatal morbidity [5, 6]. The prenatal course was further complicated by hydrops, necessitating fetal thoracocentesis, and amnioreduction at 32 weeks and one day of gestation. She was delivered via urgent cesarean section due to non-reassuring fetal heart tracing at 33 weeks and two days. The birth weight was 2,190 g. She was placed on veno-arterial ECMO within three hours after birth because of worsening hypercarbia and hypoxemic respiratory failure caused by severe pulmonary hypoplasia. The newborn was cannulated using an 8 Fr arterial cannula in the right common carotid artery and an 8 Fr venous cannula in the right internal jugular vein. She underwent primary diaphragmatic repair on day of life (DOL) eight and was successfully weaned from ECMO by day of life 15.

On DOL 152, she developed a urinary tract infection caused by Klebsiella pneumoniae. Adequate antibiotic coverage with cefotaxime was initiated and quickly broadened to vancomycin, flagyl, and cefepime; however, her condition deteriorated rapidly. This deterioration was marked by an acute increase in B-type natriuretic peptide, rising to 3,718 pg/mL, and was accompanied by biventricular dysfunction, right ventricular failure, and mitral valve stenosis on repeat echocardiogram. Despite maximal intensive medical management for severe pulmonary hypertension crisis and associated cardiac dysfunction, she developed worsening hemodynamics, further deterioration of biventricular function, and end-organ dysfunction. This prompted the decision to reinitiate venoarterial ECMO as a bridge to recovery. Transthoracic cardiac cannulation via sternotomy was urgently performed on DOL 158, as ligation of the neck vessels was performed at the time of the neonatal ECMO decannulation. A 10-French return cannula was placed in the ascending aorta, and a 20-French drainage cannula was placed in the right atrium. Her sternum was left open, but the skin was closed around the cannulas. An echocardiogram conducted on full support demonstrated improvement in biventricular function. On DOL 161, she developed oozing from the cannula site, which progressively worsened, leading to hypotension and bradycardia. A repeat echocardiogram revealed a large pericardial effusion with signs of cardiac tamponade; therefore, she underwent an urgent bedside mediastinal washout. Bleeding from the aortic cannulation site was controlled by placing additional purse-string sutures. The remaining course of the repeat ECMO was uneventful, and she was successfully decannulated on day 167. The development of a large left holo-hemispheric extra-axial, subdural multiloculated hematoma with mild mass effect in the adjacent brain parenchyma, prominence of the subarachnoid spaces, and a noticeable loss of brain volume complicated her post-decannulation course. Additionally, surveillance MRI revealed a reduction in white matter and thinning of the corpus callosum with under-opercularization.

Following her second ECMO course, she had ongoing ventilator-dependent respiratory insufficiency, which resulted in the placement of a tracheostomy on DOL 261 after several unsuccessful extubation attempts. Additionally, she experienced failure to thrive, oral aversion, and gastroesophageal reflux disease, leading to gastrojejunostomy placement on DOL 223. Her pulmonary hypertension was managed with inhaled nitric oxide, Sildenafil, and Bosentan. She was eventually discharged to a long-term care facility on DOL 378 for management of persistent pulmonary hypertension, tracheostomy/ventilator-dependent respiratory insufficiency, and feeding intolerance. Additionally, her post-discharge course has been complicated by bilateral conductive hearing loss, hypotonia, and global developmental delay. A formal neurodevelopmental evaluation was conducted at 19 months of age using the Bayley Scales of Infant Development, 3rd Edition. Her cognitive, language, and motor skills are severely delayed, with composite scores of 55, 63, and 58, respectively. She now lives at home with healthcare support. She was eventually weaned off the ventilator during the day, achieved full enteral gastrojejunostomy feeds, and her pulmonary hypertension is managed with Tadalafil and Ambrisentan. She is participating in physical, occupational, and speech therapy and early intervention.

## Discussion

To improve decision-making regarding multi-course ECMO in patients with CDH, it is essential to better understand the potential survival benefits, risks, and surgical difficulties associated with this approach. The need for repeat ECMO is rare. Over the past decade, 458 neonates with CDH have been cared for at the Children’s Hospital of Philadelphia, of which 96 required a single ECMO run. As a result, the prevalence of repeat ECMO in patients who required ECMO at our institution, 1%, is even lower than the reported rate of 5.3% in the study group registry [3]. Given the rarity of repeat ECMO in CDH, establishing clear guidelines in this complex situation remains challenging. We demonstrate that repeat ECMO can be a life-saving strategy for selected CDH patients when a reversible cause for clinical deterioration can be identified. Our findings also show that recannulation at a different site is technically feasible and safe. Our case is distinct from previously reported instances because the need for repeated ECMO cannulation typically occurs within the first week after decannulation [3]. Early recannulation may indicate that the underlying condition that required the initial ECMO treatment has not fully resolved or could still be linked to the issues from the first ECMO run. In our patient, the original cause for ECMO – hypoxemic respiratory failure – had been resolved, and repeat ECMO was necessary due to an acute exacerbation of pulmonary hypertension resulting from urosepsis. We demonstrate that late-term recannulation, even months after the initial cannulation, is possible and can effectively improve management and survival in patients with CDH.

Formulating an appropriate re-cannulation strategy early on is essential. Repeated neck cannulation has been used in cases where the intervals between ECMO courses are short [3]. However, cannulating the same side after nearly five months, as in our case, was not feasible due to the ligation of the right internal jugular vein and the common carotid artery close to the clavicle. Known factors such as insufficient proximal vessel length, previous tissue trauma, scarring, and increased inflammation at the initial cannulation site also prevented us from using the same-site approach in two previously reported cases that required re-cannulation years after the initial ECMO run [7]. Dela Cruz et al. [7] have proposed that initial cannulation should be performed in the midsection of the neck to provide adequate proximal vessel length in case re-cannulation becomes necessary. Cannulating the contralateral neck would have led to occlusion of both carotid arteries and jugular veins, potentially compromising arterial brain perfusion and venous return. In our case, femoral vessel cannulation was not possible due to the patient’s size, the risk of ipsilateral limb ischemia, and the risk of “north-south” syndrome. Therefore, we chose central cannulation via the aorta and right atrium. In addition to the anatomical considerations, central ECMO cannulation appeared to be an appropriate alternative, as it has been shown to improve survival in children with refractory septic shock [8].

While multiple courses of ECMO may provide potential survival benefits, these advantages must be balanced against the increased risk of complications. Survivors of congenital diaphragmatic hernia (CDH) who require repeat ECMO cannulation face the highest rates of morbidity [3]. In our case, we saw several short- and long-term complications, including substantial respiratory and gastrointestinal problems. We also observed significant neuroimaging abnormalities and severe neurodevelopmental impairments. Given that both prematurity and ECMO use are linked to adverse neurological outcomes in CDH [9, 10], it remains uncertain whether the increased risk of multisystem morbidities stems from the severity of the disease necessitating repeat ECMO, the treatment itself, or a combination of both factors. These morbidities, however, should not necessarily prevent repeat ECMO use but rather guide the discussions with families and emphasize the importance of close follow-up for these patients.

Our patient recovered from an acute exacerbation of pulmonary hypertension, demonstrating that repeat ECMO is not futile in this clinical scenario. Successful recannulation requires multidisciplinary discussions and excellent collaboration and coordination among medical and surgical teams. However, due to the lack of selection criteria and the high likelihood of increased multisystem morbidities, this intensified approach should be highly individualized.

## Data Availability

The data supporting this study’s findings are available from the corresponding author upon reasonable request.

## References

[1] Danzer E, Hedrick HL. Controversies in the management of severe congenital diaphragmatic hernia. Semin Fetal Neonatal Med. 2014;19:376–384. 25454678 10.1016/j.siny.2014.10.001

[2] Jancelewicz T, Langham MR Jr., Brindle ME, et al. Survival benefit associated with the use of extracorporeal life support for neonates with congenital diaphragmatic hernia. Ann Surg. 2022;275:e256–e263. 33060376 10.1097/SLA.0000000000003928

[3] Danzer E, Harting MT, Dahlen A, et al. Impact of repeat extracorporeal life support on mortality and short-term in-hospital morbidities in neonates with congenital diaphragmatic hernia. Ann Surg. 2023;278:e605–e613. 36102187 10.1097/SLA.0000000000005706

[4] Meyers ML, Garcia JR, Blough KL, et al. Fetal lung volumes by mri: normal weekly values from 18 through 38 weeks’ gestation. Am J Roentgenol. 2018;211:432–438. 29894217 10.2214/AJR.17.19469

[5] Hedrick HL, Danzer E, Merchant AM, et al. Liver position and lung-to-head ratio for prediction of extracorporeal membrane oxygenation and survival in isolated left congenital diaphragmatic hernia. Am J Obstet Gynecol. 2007;197:e421–e424. 10.1016/j.ajog.2007.07.001 17904987

[6] Victoria T, Danzer E, Adzick NS. Use of ultrasound and MRI for evaluation of lung volumes in fetuses with isolated left congenital diaphragmatic hernia. Semin Pediatr Surg. 2013;22:30–36. 23395143 10.1053/j.sempedsurg.2012.10.006

[7] Dela Cruz TV, Stewart DL, Robinson TW, et al. The use of a second course of extracorporeal membrane oxygenation in neonatal patients. ASAIO J. 1996;42:230–232. 8725696

[8] MacLaren G, Butt W, Best D, et al. Central extracorporeal membrane oxygenation for refractory pediatric septic shock. Pediatr Crit Care Med. 2011;12:133–136. 20453704 10.1097/PCC.0b013e3181e2a4a1

[9] Danzer E, Gerdes M, D’Agostino JA, et al. Younger gestational age is associated with increased risk of adverse neurodevelopmental outcome during infancy in congenital diaphragmatic hernia. J Pediatr Surg. 2016;51:1084–1090. 26831532 10.1016/j.jpedsurg.2015.12.010

[10] Danzer E, Hoffman C, D’Agostino JA, et al. Short-term neurodevelopmental outcome in congenital diaphragmatic hernia the impact of extracorporeal membrane oxygenation and timing of repair. Pediatr Crit Care Med. 2018;19:64–74. 29303891 10.1097/PCC.0000000000001406

